# IL-1β Processing in Host Defense: Beyond the Inflammasomes

**DOI:** 10.1371/journal.ppat.1000661

**Published:** 2010-02-26

**Authors:** Mihai G. Netea, Anna Simon, Frank van de Veerdonk, Bart-Jan Kullberg, Jos W. M. Van der Meer, Leo A. B. Joosten

**Affiliations:** Department of Medicine, Radboud University Nijmegen Medical Center, and Nijmegen Center for Infections, Inflammation and Immunity (N4i), Nijmegen, The Netherlands; University of California San Diego, United States of America

## Abstract

Stimulation and release of proinflammatory cytokines is an essential step for the activation of an effective innate host defense, and subsequently for the modulation of adaptive immune responses. Interleukin-1β (IL-1β) and IL-18 are important proinflammatory cytokines that on the one hand activate monocytes, macropages, and neutrophils, and on the other hand induce Th1 and Th17 adaptive cellular responses. They are secreted as inactive precursors, and the processing of pro-IL-1β and pro-IL-18 depends on cleavage by proteases. One of the most important of these enzymes is caspase-1, which in turn is activated by several protein platforms called the inflammasomes. Inflammasome activation differs in various cell types, and knock-out mice defective in either caspase-1 or inflammasome components have an increased susceptibility to several types of infections. However, in other infections and in models of sterile inflammation, caspase-1 seems to be less important, and alternative mechanisms such as neutrophil-derived serine proteases or proteases released from microbial pathogens can process and activate IL-1β. In conclusion, IL-1β/IL-18 processing during infection is a complex process in which the inflammasomes are only one of several activation mechanisms.

## The Role of IL-1β and IL-18 in Host Defense

The main cellular innate host defense mechanisms are the phagocytosis and killing of bacteria and fungi by neutrophilic granulocytes, monocytes, and macrophages [1],[2], and the lysis of viral-infected cells by natural killer (NK) cells [3]. Upon recognition of a microorganism, proinflammatory cytokines such as tumor necrosis factor (TNF), interferon-γ (IFNγ), interleukin (IL)-18, and IL-1β are secreted. These cytokines activate neutrophils and macrophages to phagocytose the invading pathogen and to release toxic oxygen and nitrogen radicals. TNF is an essential component of the host defense, as demonstrated by the important infectious complications in patients treated with anti-TNF biological agents [4]. Similarly, IFNγ activates both neutrophils and macrophages for intracellular killing of bacteria or fungi. Patients with defects in the IL-12/IFNγ activation pathways are at increased risk of severe mycobacterial and *Salmonella* infections [5], and recombinant IFNγ is an established therapy in patients with chronic granulomatous disease [6]. However, in addition to TNF and IFNγ, the proinflammatory cytokines of the IL-1 family, most notably IL-1β and IL-18, also have very important roles for antimicrobial host defense. IL-1α and IL-1β, which bind and activate the same receptor [7], activate the release of other proinflammatory cytokines such as TNF and IL-6, and induce a Th17 bias in the cellular adaptive responses [8]. In vivo, IL-1 is largely responsible for the acute phase response, which includes fever, acute protein synthesis, anorexia, and somnolence [7], while IL-18 is essential for the induction of IFNγ and Th1 responses [9]. Through these mechanisms, cytokines of the IL-1 family are a crucial component of the host defense against infections.

## IL-1β and IL-18 Processing and Release: The Inflammasomes

Much interest has been generated regarding the processing and release of bioactive IL-1β since the discovery of an entire group of disorders called autoinflammatory syndromes that specifically respond to the blockade of the IL-1 receptor with the IL-1 receptor antagonist (IL-1Ra), or with neutralization of IL-1β by the monoclonal anti-IL-1β antibodies. These syndromes are characterized by attacks of sterile inflammation of joints, serositis, fever, and skin lesions. Some of the best known diseases in this group include familial Mediterranean fever (FMF) [10], cryopyrin-associated periodic syndromes (also known as cryopyrinopathies, which include familial cold auto-inflammatory syndrome [FCAS] [11], Muckle-Wells syndrome [MWS] [12], and neonatal onset multisystem inflammatory disease [NOMID] [13]), hyperimmunoglobulin D syndrome (HIDS) [14], TNF receptor–associated periodic syndrome (TRAPS), and adult-onset Still's disease [15]. Blood monocytes from patients with some of these disorders, especially cryopyrinopathies, readily release more IL-1β than monocytes from unaffected controls, revealing a loss of the tight control that regulates the processing and release of active IL-1β. An abnormal production of IL-1β has been therefore proposed to be the underlying cause of these diseases.

Several mechanisms control the production and activity of IL-1β, including the processing of the 31-kDa inactive IL-1β precursor form into the bioactive 17-kDa IL-1β [16], and the release from secretory lysosomes through K^+^-dependent mechanisms [17],[18]. In addition, control over IL-1 activity is exerted by the IL-1 receptor antagonist (IL-1Ra) or the type II decoy receptors [19]. Processing of bioactive IL-1β (and that of IL-18) depends on activation of caspase-1 by protein complexes termed the inflammasomes [20]. Several protein platforms/inflammasomes have been described for the activation of caspase-1, and each of them include members of the NOD-like receptor (NLR) family of proteins [21]. Through CARD–CARD and pyrin domain–pyrin domain interactions, a large macromolecular complex is formed to represent a scaffold for the recruitment and activation of pro-caspase-1. It is believed, yet not proven, that caspase-1 activation in the inflammasome is induced by the formation of oligomers and proximity between caspase-1 molecules.

**Figure 1:**
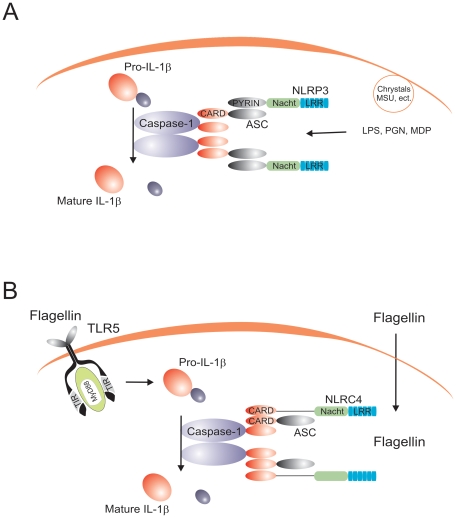
The prototypical NLRP3 and NLRC4 inflammasomes. (A) The NLRP3 inflammasome is activated by both bacterial (e.g., MDP, bacterial RNA, β-glucan), exogenous (e.g., silica, alum), and endogenous (e.g., uric acid cristals, ATP) stimuli. (B) The NLRC4 inflammasome is activated by flagellin in a TLR5-independent fashion.

Several major inflammasome complexes that activate caspase-1 have been described to date. The most intensely studied has been the inflammasome formed by the NLR family member NLRP3, which forms complexes that include the adapter protein ASC for the activation of caspase-1 (Figure 1A). Mutations in NLRP3 have been described in the cryopyrin-associated periodic syndromes (CAPS; cryopyrin is a name previously used for NLRP3), whereas specific NLRP-3 polymorphisms have been associated with Crohn's disease [22]. A large number of stimuli have been described to activate the NLRP3 inflammasome: some of them of bacterial origin (muramyl dipeptide [MDP], bacterial RNA, double-stranded RNA), some of them are danger-associated molecular patterns (uric acid crystals, amyloid-β), but also exogenous compounds such as asbestos, silica, or alum adjuvant [23]–[27]. The precise mechanism leading to the activation of the NLRP3 is still unclear. The diverse molecular structure of these compounds most likely precludes the direct stimulation of the NLRP3 inflammasome. A unifying hypothesis proposes that common intracellular activities such as induction of hypokalemia, reactive oxygen species, or calcium-dependent phospholipase 2 are indirectly activating the inflammasome [28]. However, stimulation of cells solely with ATP, a known inducer of potassium efflux through P2X7-mediated mechanisms, is unable to activate caspase-1, and cell priming with lipopolysaccharide (LPS) is necessary for ATP to induce inflammasome activation. In this context, induction of NF-kB-dependent transcription of NLRP3 by Toll-like receptor (TLR) ligands [29] or proinflammatory cytokines [30] seems to be the critical checkpoint needed for cell priming prior to inflammasome activation by ATP. In addition, formation of pores by pannexin-1 is one mechanism through which microbial products (e.g., MDP) can be delivered into the cytoplasm to activate the inflammasome [31].

The only inflammasome that has been reconstituted biochemically is the NLRP1 inflammasome. A study using purified NLRP1, ASC, and caspase-1 has shown that NLPR1 forms oligomers in the presence of MDP [32]. However, no evidence has been presented that MDP can actually bind NLRP1, although another study suggested the involvement of NOD2/NLRP1 complexes in this process [33]. NLRP-1 polymorphisms have been associated with vitiligo and autoimmune diseases [34].

In addition to the NLRPs, another NLR member, NLRC4/IPAF, forms an inflammasome that activates caspase-1 in response to intracellular flagellin in an ASC-independent manner [35],[36]. Caspase-1 activation by flagellin/NLRC4 is independent of TLR5, suggesting that flagellin recognition is mediated by two systems: extracellular sensing by TLR5, and intracytoplasmatic sensing by NLRC4 (Figure 1B). Finally, a newly described mechanism involving recognition of bacterial DNA by the intracellular sensor AIM2 suggests the existence of a specific inflammasome complex that induces caspase-1 activation upon sensing nucleic acids [37],[38]. This is a particularly important finding, as intracellular detection of DNA from invading pathogens is likely to be central for an effective host defense. AIM2-dependent activation of IL-1β has been suggested to be critical for the activation of host defense against vaccinia virus and *Francisella tularensis* [37], and it is to be expected that similar effects will be identified in the future for other pathogens.

Through these studies on the structure and function of the various inflammasomes, a dogma has emerged during the last few years in which production and release of IL-1β and IL-18 is the result of two independent signals: one signal is induced through pattern recognition receptors ( e.g., TLRs) to activate transcription of pro-IL-1β and pro-IL-18, and one signal is mediated by the NLR-containing inflammasomes (and independent of the TLRs) that induce cleavage of cytokine precursors into the active IL-1β and IL-18 forms through caspase-1 activation.

## Differential Role of the Inflammasome in Monocytes and Macrophages

Despite the progress made in understanding the process of IL-1β synthesis, controversy surrounded the capacity of TLR ligands such as LPS to activate caspase-1 and cause the release of active IL-1β. By using transfected cell lines and/or NLRP3 knock-out mice, a broad panel of exogenous and endogenous stimuli have been proposed to activate the NLRP3 inflammasome (see above), but purified TLR ligands such as LPS were not among these inflammasome stimuli. Therefore, based on defective responses of the monocyte-like leukemia cell line THP-1 to LPS stimulation, a concept has arisen that IL-1β production induced by LPS is due to contamination with non-LPS ligands such as peptidoglycans [23], while LPS by itself is ineffective as a stimulator of IL-1β release. A second signal, such as MDP or ATP, is required, and this would induce activation of caspase-1 followed by IL-1β processing and release [39]. However, this model is derived from data in THP-1 cells [23] and in primary mouse macrophages [31], and it is inconsistent with many studies showing abundant production and release of IL-1β from blood monocytes by TLR ligands such as purified LPS, lipopeptides, and lipoteichoic acid, as well as cytokines such as TNFα and IL-1 itself [40],[41]. In addition, several studies reported that synthetic products, which exclude contamination with NLRP1 or NLRP3 ligands, stimulate IL-1β release [42],[43].

**Figure 2:**
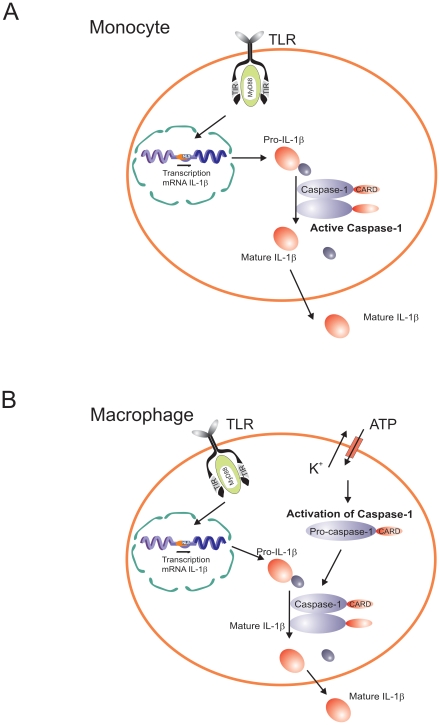
Diagram representing the differential caspase-1/IL-1β activation pathways in monocytes and macrophages. Caspase-1 is constitutively activated in monocytes, and these cells release mature IL-1β after single stimulation with TLR ligands. IL-1β secretion is induced by endogenously-released ATP. In contrast, macrophages need a double stimulation: one stimulus (TLR-ligands) induces transcription, and a second stimulus (ATP) induces IL-1β secretion.

These apparent discrepancies have been resolved by a study from our group showing that synthesis and release of IL-1β differ between human monocytes and macrophages. Monocytes have constitutively activated caspase-1, leading to release of active IL-1β after a single stimulation event with bacterial ligands such as LPS, whereas macrophages (and THP-1 cells) need two distinct stimuli: one stimulus induces transcription and translation, and a second stimulus is needed for caspase-1 activation with subsequent IL-1β processing and secretion [44] (Figure 2). Although caspase-1 is constitutively activated in human monocytes, that is still dependent on inflammasome components, as the inhibition of ASC by siRNA results in a significant reduction of both caspase-1 activation and processing of IL-1β [44]. A crucial functional aspect in relation to the constitutive inflammasome activation in monocytes relates to the release of endogenous ATP by monocytes. Endogenous ATP from monocytes can in turn activate the NLRP3 inflammasome and induce IL-1β secretion through P2X7. In contrast, macrophages completely lack the capacity to release ATP [45].

Consistent with the failure of in vitro–differentiated macrophages to release IL-1β is the long known defect in IL-1β synthesis of the alveolar macrophages. Wewers and colleagues proposed a post-transcriptional defect in freshly obtained alveolar macrophages [46]. Recently, they reported differences in pyrin expression between monocytes and macrophages, and suggested that pyrin induces IL-1β release [47]. Monocytes from patients with FMF who have mutations in pyrin release more IL-1β upon stimulation than cells from control subjects, suggesting a failure to suppress the activation of caspase-1 [10].

These data imply a paradigm shift in our understanding of the inflammasome. The demonstration of a role for ASC and NLRP3 in the constitutive activation of caspase-1, independent of stimulation by TLRs or inflammasome ligands, uncouples caspase-1 activation from pathogen-associated molecular pattern (PAMP) recognition in human primary monocytes. This new model, in which the inflammasome components ASC and NLRP3 form a protein platform responsible for the constitutive activation of caspase-1, explains why IL-1β induction in monocytes by a very diverse panel of stimuli (including TLR ligands) is caspase-1 dependent, although these stimuli need not themselves be involved in inflammasome activation. In addition, a role of ASC and NLRP3 in caspase-1 activation in the monocyte, independently of “classical” inflammasome stimuli, explains the resistance to experimental endotoxemia in ASC−/− and NLRP-3−/− mice [48],[49]. In contrast, macrophages need two signals in order to produce IL-1β, in a model close to the current concept in the literature: one signal is mediated by TLRs to induce gene transcription, and a second signal to induce inflammasome activation for the processing of IL-1β.

The single (TLR ligand only) stimulation in monocytes compared with the double (TLR ligand/ATP) stimulation in macrophages (Figure 2) likely represents an adaptation of each cell type to their respective environments. Circulating monocytes function in the surveillance of an essentially pathogen-free environment, so they must respond promptly to any danger signal (especially of microbial origin). On the other hand, macrophages are confined to an environment (e.g., alveolar space, mucosal surfaces) in which they are constantly exposed to (colonizing) microbial stimuli. An easily inducible response of macrophages to release IL-1β for each encounter with such exogenous stimuli would result in chronic deleterious inflammatory reactions. Thus, repeated bouts of inflammation are likely reduced by the requirement of a second stimulus for the activation of the inflammasome and release of active IL-1β. Such second stimuli would be available at sites of infection, trauma, or necrosis where ATP levels are elevated and can trigger the P2X7 receptor [50]. In addition, second signals can come from the cathelicidin-derived peptide LL37 from infiltrating neutrophils [51], or the release of bacterial toxins [25]. One situation in which caspase-1 activation seems to be constitutively activated also in macrophages is represented by the absence of the autophagy gene ATG16L1, although the precise mechanism responsible for this effect is not yet elucidated [52]. However, the association of ATG16L1 polymorphisms with Crohn's disease makes this aspect potentially important for the pathophysiology of this important disease [53],[54].

## The Role of Caspase-1 and the Inflammasome in Antimicrobial Host Defense

Due to their role in the processing of IL-1β and IL-18, caspase-1 and the inflammasome components are bound to have important roles in the host defense against pathogenic microorganisms. In vitro studies have shown that *Bacillus anthracis* toxin activates IL-1β through the *Nalp1b* inflammasome in the mouse [55]. Similarly, NLRP3 activation of caspase-1 has been linked to a variety of microorganisms ranging from the bacteria *Listeria monocytogenes* [56],[57] and *Staphylococcus aureus* [58] to viruses such as influenza, adenoviruses, and Sendai virus [59]. The interaction of NLRC4 with microorganisms was probably one of the best characterized, demonstrating the involvement of NLRC4 (independently of ASC) in the activation of caspase-1 by *Salmonella typhimurium* [35],[36],[48], *Legionella pneumophila* [60], *Pseudomonas aeruginosa* [61],[62], and *Shigella flexneri* [63].

In vivo experimental models of infection have also demonstrated that the lack of caspase-1 in knock-out mice leads to an increased susceptibility to a variety of infections, including those with *F. tularensis* [64], *L. pneumophila* [65], *Shigella* [63], *Salmonella* [66],[67], and *P. aeruginosa* [62] (Table 1). What all of these infections have in common is that caspase-1 activity, and thus IL-1β production, is dependent on the assembly of an inflammasome complex [20], although they may differ in their specific inflammasome components. Experimental infections with some of these pathogens have been also investigated in knock-out mice lacking components of the inflammasome. In this respect, ASC-deficient mice have been shown to be more susceptible to infections with some bacteria (*Francisella* and *Staphylococcus*) [64],[68], as well as to influenza viruses [69], demonstrating its importance in host defense mechanisms. As in some models ASC−/− mice are clearly more susceptible to infections than NLRP3−/− mice (e.g., influenza [69]), one may suggest a more important role for ASC in antimicrobial defense. Alternatively, the partial redundancy between different NLRs may explain the more pronounced phenotype of ASC knock-out mice compared to single NLR-deficient mice.

**Table 1:** Susceptibility to In Vivo Experimental Models of Infection in Mice Deficient in IL-1β, IL-18, or Inflammasome Components.

	IL-1β−/−	IL-18−/−	Caspase-1−/−	NLRP3−/−	ASC−/−	NLRC4−/−
**Endotoxemia**	Normal [74]	Lower [95],[96]	Lower [72],[73]	Lower [49]	Lower [48],[49]	ND
**Turpentine**	Lower [71],[74]	ND	Normal [70]	ND	ND	ND
***E. coli***	Normal [97]	Normal [97]	Higher [98]; Lower [97]	ND	ND	ND
***Shigella***	Higher [99]	Higher [99]	Higher [99]	ND	ND	ND
***Salmonella***	Higher [66]	Higher [66]	Higher [66]	Normal [67]	Normal [67]	Normal [67]
***C. albicans***	Higher [77],[87]	Higher [100]	Normal [78]; Higher [80]	Higher [79],[80]	ND	ND
***S. aureus***	Higher [68]	Higher [101]	ND	ND	Higher [68]	ND
***C. trachomatis***	ND	Normal [102]	Normal [76],[102]	ND	ND	ND
***Listeria***	Normal [103]	Lower [104]	Higher [105]	ND	ND	ND
***Francisella***	ND	ND	Higher [64]	ND	Higher [64]	ND
***Legionella***	ND	ND	Higher [106]	ND	ND	Higher [60],[106]
***Influenza***	Higher [69]	Lower [107]; Higher [108]	Higher [69]	Normal [69]	Higher [69]	ND

Lower (susceptibility): increased survival of the knock-out mice in the experimental model.

Higher (susceptibility): decreased survival of the knock-out mice in the experimental model.

ND, not done.

## Inflammasome-Independent IL-1β Activation

Despite the importance of inflammasome activation in certain experimental models of inflammation, certain in vivo infection models in mice deficient in inflammasome components show intriguing results that question the importance of the inflammasome (Table 1). One category of results shows that although IL-1β is definitely important for inflammatory reactions in antimicrobial defense, components of the inflammasome or even caspase-1 can be dispensable. In models of sterile inflammation induced by turpentine, IL-1β−/− mice are protected against inflammation, while caspase-1−/− mice are not [70],[71]. This is in clear contrast with LPS models in which caspase-1−/− mice are protected (Table 1) [72],[73]. It appears therefore that caspase-1 and inflammasome activation is important in some, but not all, types of IL-1β-driven inflammation [74]. Furthermore, caspase-1 seems not to be involved in the host defense against certain types of microorganisms such as *Chlamydia trachomatis* [75],[76], although IL-1β is involved in the inflammatory responses induced by these microorganisms [77]. These data argue for inflammasome-independent activation of IL-1β in certain infectious processes.

An interesting case regarding the involvement of the inflammasome in host defense is represented by the fungal pathogen *Candida albicans*. IL-1 plays an important role for the host defense and survival of mice during disseminated candidiasis [75],[85]. Surprisingly, caspase-1-deficient mice have been reported to have a normal resistance to disseminated candidiasis [78], suggesting activation of IL-1β by alternative mechanisms (see below). However, NLRP3−/− and ASC−/− mice have been reported to be more susceptible to both systemic [79] and mucosal [80] *Candida* infections, opening the intriguing possibility of biological functions of inflammasome components that are not related to caspase-1 activation. Indeed, an earlier study on the function of ASC has reported its interaction with NF-κB and an influence on gene transcription [81]. Whether ASC or NLRP3 have underestimated roles that are independent of inflammasome activation remains to be studied. These studies have shown a role of inflammasome components in experimental models of *Candida* infection in mice, and in line with this the activation of IL-1β in human monocytes is dependent on caspase-1. However, in contrast to mouse macrophages, caspase-1 is constitutively activated in human monocytes and thus does not need fungal recognition by the NLRs in the inflammasome [82].

## Alternative Mechanisms of IL-1β Processing

**Figure 3:**
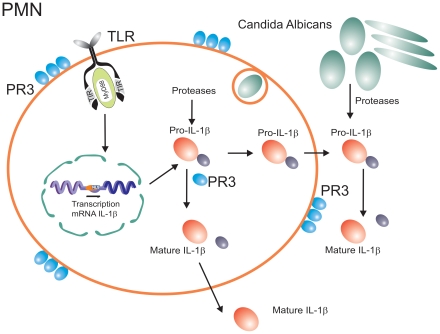
Inflammasome-independent processing of pro-IL-1β. In addition to caspase-1-dependent activation, pro-IL-1β can also be processed by neutrophil-derived serine proteases, or pathogen-derived proteases.

Shortly after the discovery of IL-1β, when it was apparent that cleavage of the pro-cytokine is needed for activation, a question arose as to whether other enzymes apart from caspase-1 would also be capable of processing pro-IL-1β. Indeed, subsequent studies have identified neutrophil- and macrophage-derived serine proteases such as proteinase-3 (PR3), elastase, and cathepsin-G as enzymes that can process pro-IL-1β into 21-kDa active fragments [7],[83]. Similarly, processing of pro-IL-18 by PR3 can also lead to active fragments [84]. The crucial role played by neutrophil-dependent, inflammasome-independent activation of pro-IL-1β has been elegantly confirmed recently by the group of Greten and colleagues [85] (Figure 3).

The inflammasome-independent activation of pro-IL-1β in situations when neutrophils are the major cell population in the inflammatory infiltrate can explain many of the apparently puzzling observations reviewed above. It is the neutrophils that form the backbone of the inflammatory infiltrates during disseminated candidiasis [86], and this explains the dependency of host defense against *Candida* on IL-1β [87], most likely activated by neutrophil-derived PR3, rather than caspase-1 [78]. Similarly, inflammatory infiltrates during arthritis consist of both macrophages and neutrophils. Indeed, we have observed a minimal role of caspase-1 during the acute inflammation of arthritis that is characterized by an overwhelming neutrophil infiltrate. In this phase of the inflammation, serine proteases such as PR3 played a more important role [88]. In contrast, during the chronic phase of inflammation when macrophages are the main component of the infiltrate, caspase-1 seems to have a more significant effect [88].

Neutrophil-derived serine proteases are not the only alternative mechanism of activation of pro-IL-1β. One very interesting phenomenon has recently been reported during infection with *C. albicans*, in which a fungus-derived protease can lead to processing and activation of host-derived pro-IL-1β and thus activation of the immune system [89]. This may represent an indirect pathway of *Candida* recognition by the innate immune system (Figure 3), reminiscent of the detection of the fungus *Beauveria bassiana* in *Drosophila* through maturation of the protease *Persephone* by a fungal-derived enzyme, leading to Toll activation [90]. Considering the production of a vast array of proteases by practically all pathogenic microorganisms, it is to be expected that similar IL-1β activation pathways are active during other infections.

## Conclusions and Perspectives: IL-1β Processing beyond the Inflammasomes

A wealth of information regarding the mechanisms of pathogen recognition and activation of innate immunity has been accumulated during the last few years, and has contributed greatly to a better understanding of the host defense against pathogenic microorganisms. One of the most exciting areas of advancement was represented by the description of the inflammasomes and the mechanisms that lead to the processing and activation of cytokines of the IL-1 family.

There is no doubt that these discoveries have contributed to a better understanding of inflammatory processes. Beautifully designed studies have also shown that caspase-1-dependent mechanisms of IL-1β and IL-18 activation have important consequences during inflammation. However, as this review has tried to bring to light, the role played by the inflammasomes should not deter the acknowledgement of other mechanisms leading to IL-1β processing that may be just as important.

As shown above, inflammasome activation is not the same in all cell types, and caspase-1 activation is not the only mechanism leading to the processing of pro-IL-1β into active fragments. Neutrophil-derived serine proteases and pathogen-released enzymes can also process and activate IL-1β, and these processes have important effects during inflammation and infection. Further characterization of these alternative mechanisms can lead to the design of improved therapeutic strategies. In this respect, any inflammatory condition in which neutrophils are involved (e.g., rheumatoid arthritis, Crohn's disease, or gout) is unlikely to respond to caspase-1 inhibition alone, and a combination of caspase-1 and PR3 inactivation may be necessary. Beneficial therapeutic effects of the IL-1 receptor antagonist (IL-1Ra, anakinra) have often been presented as proof-of-principle for a role of the inflammasome in certain clinical conditions such as gout [91],[92], in which neutrophils play a crucial role [93]. This assumption is clearly too preliminary: IL-1Ra will block the effects of IL-1β irrespective of the mechanisms that led to its activation, apart from the fact that IL-1α effects are also blocked.

A similar generalization based on IL-1Ra effects is currently the dogma regarding the pathophysiology of autoinflammatory syndromes: many of the autoinflammatory syndromes are considered defects of inflammasome activation. While this is likely the case for some diseases, for example CAPS, in which NLRP3 mutations are the cause of the disease [94], this relation is much less clear in other autoinflammatory disorders. It is better to consider the disorders in which anakinra is active as “IL-1Ra responsive diseases” or perhaps “IL-1 related diseases” instead of immediately considering them “inflammasome-related diseases”. While caspase-1 is an obvious candidate to be investigated, neglecting to investigate other IL-1β-activating mechanisms (or IL-1α secretion) would be an oversight.

To conclude, the description of the inflammasomes has taught us a lot, but we should not fall in the trap of our own success. Caspase-1 activation is just one of the mechanisms of activation of one of the two active IL-1 molecules, and it is unlikely that an entire class of PAMP recognition receptors (the NLR receptors) have evolved only to be devoted to a single immunological function. While acknowledging the importance of the inflammasomes for our understanding of inflammatory reactions, we should consider in our endeavors the alternative pathways of IL-1β/IL-18 activation, and also actively examine alternative roles of the NLR class of receptors.
